# Interaction of *Arsenophonus* with *Wolbachia* in *Nilaparvata lugens*

**DOI:** 10.1186/s12862-021-01766-0

**Published:** 2021-02-20

**Authors:** Huifang Guo, Na Wang, Hongtao Niu, Dongxiao Zhao, Zhichun Zhang

**Affiliations:** Institute of Plant Protection, Jiangsu Academy of Agricultural Sciences, No.50, Zhongling street, Nanjing, 210014 Jiangsu China

**Keywords:** *Nilaparvata lugens*, *Wolbachia*, *Arsenophonus*, Co-infection

## Abstract

**Background:**

Co-infection of endosymbionts in the same host is ubiquitous, and the interactions of the most common symbiont *Wolbachia* with other symbionts, including *Spiroplasma*, in invertebrate organisms have received increasing attention. However, the interactions between *Wolbachia* and *Arsenophonus,* another widely distributed symbiont in nature, are poorly understood. We tested the co-infection of *Wolbachia* and *Arsenophonus* in different populations of *Nilaparvata lugens* and investigated whether co-infection affected the population size of the symbionts in their host.

**Results:**

A significant difference was observed in the co-infection incidence of *Wolbachia* and *Arsenophonus* among 5 populations of *N. lugens* from China, with nearly half of the individuals in the Zhenjiang population harbouring the two symbionts simultaneously, and the rate of occurrence was significantly higher than that of the other 4 populations. The *Arsenophonus* density in the superinfection line was significantly higher only in the Maanshan population compared with that of the single-infection line. Differences in the density of *Wolbachia* and *Arsenophonus* were found in all the tested double-infection lines, and the dominant symbiont species varied with the population only in the Nanjing population, with *Arsenophonus* the overall dominant symbiont.

**Conclusions:**

*Wolbachia* and *Arsenophonus* could coexist in *N. lugens*, and the co-infection incidence varied with the geographic populations. Antagonistic interactions were not observed between *Arsenophonus* and *Wolbachia*, and the latter was the dominant symbiont in most populations.

## Background

Symbiotic associations between prokaryotic and eukaryotic organisms are ubiquitous in natural communities, and bacterial symbiosis has played a fundamental role in the evolution of eukaryotes, which range from parasitism to mutualism [2, 20, 32]. Many invertebrate hosts have been found to harbour multiple inherited symbionts within a single host [21, 27, 30, 34, 40]. Other than co-infection of different symbiont species, co-infections with multiple strains of the same symbiont species have also been found [7, 28].

*Wolbachia* is an intracellular symbiont that infects between 20 and 76% of arthropod species [14, 38]. *Wolbachia* has been known to coinfect and interact with various symbionts in the same host, and the superinfections vary with the species of symbionts and are also affected by many other factors, including the species of insect host, environmental conditions, etc. [7, 8, 21, 28]. In *Bemisia tabaci*, *Wolbachia* was found to be present with *Hamiltonella* or *Cardinium* or both genera [8]. Co-infection of *Wolbachia* and *Cardinium* was also found in *Encarsia inaron* [39], and superinfection with combination of *Wolbachia* and *Spiroplasma* occurs in *Drosophila melanogaster*, whereas an asymmetrical interaction occurs between *Wolbachia* and *Spiroplasma* in which the population of *Wolbachia* organisms is negatively affected by *Spiroplasma* organisms while the population of *Spiroplasma* organisms is not influenced by *Wolbachia* organisms [7]. The genus *Arsenophonus* is an emerging clade of symbiotic bacteria with a vast host distribution that includes parasitic wasps, triatomine bugs, psyllids, whiteflies, aphids, ticks, planthoppers, etc. [6, 8, 10, 24, 26, 35]. However, interactions among *Arsenophonus* and *Wolbachia* are poorly understood.

Brown planthopper *Nilaparvata lugens* Stål (Homoptera: Delphacidae) is a monophagous insect herbivore of rice that causes serious damage to rice crops. *N. lugens* has been known to harbour symbionts, including *Wolbachia* and *Arsenophonus*, and previous detection has shown that although *Wolbachia* and *Arsenophonus* were present in all 15 brown planthopper populations collected from China and Southeast Asian countries, coexistence was not observed in the same individuals from Laos [26]. In this study, we investigated the co-infection of *Wolbachia* and *Arsenophonus* in different populations of *N. lugens* collected from 5 sites in China, and then we established a single-infected line (infected with only *Wolbachia*) and a double-infected line (infected with both *Wolbachia* and *Arsenophonus*). Subsequently, we examined *Wolbachia* and *Arsenophonus* titres in the double- and single-infected *N. lugens* to assess whether these two symbionts interacted mutually or competitively.

## Methods

### Field collection of *Nilaparvata lugens*

All geographic populations of brown planthopper were collected from rice paddies in different locations of China. The details of each population were listed in Table 1. The planthoppers were maintained on rice seedlings at a constant temperature of 27 (± 1) °C and a light period of 14:10 h light:dark.

**Table 1:** Information for the different geographic populations of brown planthopper

Abbreviations	Collection site	Longitude	Latitude	Collection time
NN	Nanning	108° 33′	22° 84′	2014.6
MS	Maanshan	118° 37′	31° 70′	2012.8
NJ	Nanjing	118° 46′	32° 03′	2005.8
ZJ	Zhenjiang	119° 55′	32° 00′	2012.8
NT	Nantong	120° 86′	32° 01′	2013.8

### Investigation of *Wolbachia* and *Arsenophonus* infection

To compare the co-infection of *Wolbachia* and *Arsenophonus* among different geographic populations of *N. lugens*, approximately 80 (64–88) adults were randomly collected from each population for a diagnostic PCR analysis. Extraction of DNA was the same as previously described, and only DNA samples with a ratio of OD260/OD280 ranging from 1.6 to 1.9 were used for the PCR detection [18]. The presence of *Wolbachia* and *Arsenophonus* was checked as previously described (*Wolbachia*: [41], *Arsenophonus* [31]).

### Preparation of single-infected (*Wolbachia*) line and double-infected line (*Wolbachia* and *Arsenophonus*)

Geographic populations were set up as mass bred lines. The single-infected (*Wolbachia*) line and double-infected lines (*Wolbachia* and *Arsenophonus*) were developed from each geographic population of *N. lugens*. To minimize variation in the genetic background within populations, a pair of newly emerged female and male adults was randomly selected from the same population.

To ensure that only the single infection or the double infection was being considered, at first, newly emerged brown planthoppers from each line were screened for the presence of all the known symbionts in planthoppers, which consisted of *Wolbachia*, *Arsenophonus, Cardinium hertigii*, *Acinetobacter*, *Chryseobaterium*, *Serratia* and *Arthrobacter* as previously described (*Wolbachia*: [41], *Arsenophonus* [31]; *Cardinium:* [23]; *Acinetobacter:* [33]; *Chryseobaterium:* [1]; *Serratia*: [43]; *Arthrobacter*: [15]). Then female and male parents and their offspring that were only infected with *Wolbachia* or only infected with *Wolbachia* and *Arsenophonus* were kept for subsequent experiments.

### Analysis of *Wolbachia* and *Arsenophonus* density

In order to measure the density of *Wolbachia* and *Arsenophonus*, the real-time quantitative PCR was performed with an ABI StepOne Real-Time PCR System (Applied Biosystems Inc, Foster City, CA, USA)*.* For each line, a total of 10 female and male adults was collected as one sample, and the DNA was extracted with a Wizard® Genomic DNA Purification Kit (Promega, USA). The primers of *Wolbachia* and *Arsenophonus* for the reaction were as follows: (*Wsp*-F) 5′-ATGTAACTCCAGAAATCAAACTC-3′, (*Wsp*-R) 5′-GATACCAGCATCATCCTTAGC-3′; (ARS16S-F) 5′-TTCGGTCGGAACTCAAAGG-3′ (ARS16S-R) 5′-TCTGAGTTCCGCTTCCCATC-3′. The 20 µL quantitative PCR (qPCR) reaction system included 10 µL SYBR® Premix Ex Taq (Tli RNaseH Plus) (2X) (Takara, Japan), 0.4 µL forward and 0.4 µL reverse primers, 0.4 µL ROX Reference Dye, 2 µL DNA and 6.8 µL ddH_2_O. The RT-PCR program were as follows: 95 °C for 30 s, followed by 40 cycles of 95 °C for 5 s and 60 °C for 31 s, and then 95 °C for 15 s, 60 °C for 1 min, and a final step at 95 °C for 15 s. A standard curve using real-time fluorescent quantitative PCR of the *Wolbachia wsp* gene or the *Arsenophonus* ARS16S rDNA gene was performed to determine accurate *Wolbachia* or *Arsenophonus* gene copy numbers as described previously [42]. For each sample, there was three technical replicates, and for each line, there was three biological replicates.

### Statistics

The infection incidence of *Arsenophonus* and *Wolbachia* among different populations were compared using the Chi-square test, and the density of *Arsenophonus* between the double-infected line and single-infected line were compared using Student’s t test, the density of *Wolbachia* among different populations were tested by ANOVAs. IBM Statistics (SPSS 19.0) software was used for these statistical analyses.

## Results

### Co-infection of *Wolbachia* and *Arsenophonus *varies with the population of *Nilaparvata lugens*

**Fig. 1:**
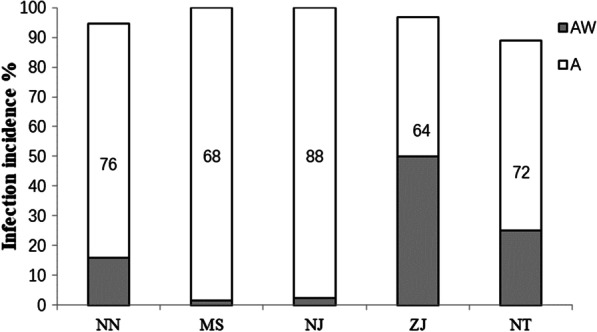
Co-infection incidences of the symbiont *Arsenophonus* and *Wolbachia* in 5 populations of *N. lugens*. A refers to all infections of *Arsenophonus* including single and double infections, and AW refers to double infections of *Arsenophonus* and *Wolbachia*. NN, MS, NJ, ZJ and NT refer to the Nanning, Maanshan, Nanjing, Zhenjiang and Nantong populations from China, respectively

The symbionts *Wolbachia* and *Arsenophonus* were detected in all the 5 populations of *N. lugens* from China (Fig. 1). Compared to *Wolbachia* infection, *Arsenophonus* infection was more common in *N. lugens*, with the infection incidence of *Arsenophonus* ranging from 88.9 to 100%. In the MS and NJ populations, all the tested individuals were infected with *Arsenophonus*, and the infection incidence was significantly higher than that in the NT population (88.9%) ($\chi_{4}^{2}$ = 17.196, *P* = 0.002, Fig. 1).

In all 5 tested populations, *Wolbachia* infection always coexisted with *Arsenophonus* infection*,* and the co-infection incidence of *Wolbachia* and *Arsenophonus* was the equivalent to the incidence of *Wolbachia* infection. The co-infection incidence in the ZJ population was 50%, which was the highest value among the 5 populations, whereas the co-infection incidence in the MS and NJ populations was rare at only 2.3% and 1.5%, respectively, while this value in the NN and NT populations was 15.5% and 25%, respectively, and a significant difference was observed among populations ($\chi_{4}^{2}$ = 75.457, *P* < 0.001, Fig. 1).

### Coexistence of *Wolbachia* does not negatively affect the density of *Arsenophonus* in most populations of *Nilaparvata lugens*

**Fig. 2:**
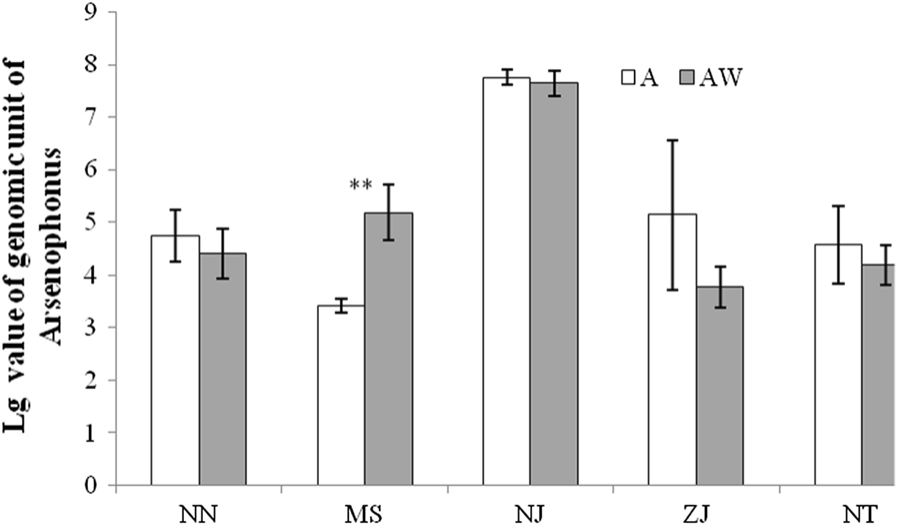
Comparison of *Arsenophonus* density in the double-infected and single-infected lines of *N. lugens*. A: single-infected line of *Arsenophonus*; and AW: double-infected line of *Arsenophonus* and *Wolbachia*. For each population, 60 adults including 30 females and 30 males were used for the analysis, and they divided into three biological replicates with 10 females and 10 males in each replicate. Significant differences between the double-infected and single-infected lines are marked by asterisks (**P < 0.01)

When *Arsenophonus* coexisted with *Wolbachia* in *N. lugens*, *Arsenophonus* density between the double-infected line and single-infected line varied based on the population (Fig. 2). In double-infected lines established from the NN, NJ, ZJ and NT populations, the *Arsenophonus* density was not significantly different from that in the single-infected lines (NN:* t* = 0*.*813, df = 4, *P* = 0*.*462; NJ:* t* = 0*.*661, df = 4, *P* = 0*.*545; ZJ:* t* = 1*.*61, df = 4, *P* = 0*.*183; NT:* t* = 0*.*803, df = 4, *P* = 0*.*467), whereas in double-infected lines established from the MS population, the *Arsenophonus* density was significantly higher than that in single-infected line (MS:* t* = 5*.*66, df = 4, *P* = 0*.*005).

### Dominance of *Wolbachia* and *Arsenophonus* varies with the population of *Nilaparvata lugens*

**Fig. 3:**
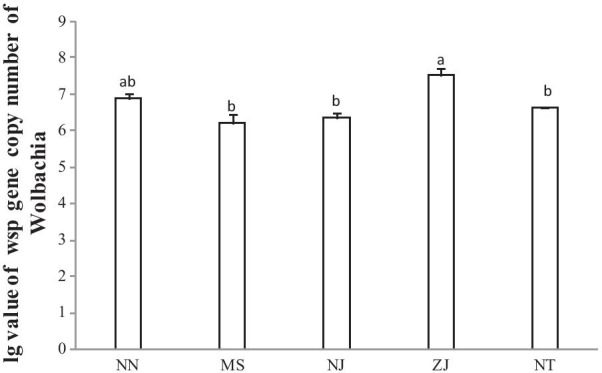
Comparison of the *Wolbachia* density in the superinfection lines of *N. lugens* established from 5 populations. There are three biological replicates, and 10 females and 10 males were used in each replicate. Values were the mean ± SE (n = 3). The same lower-case letter above the bars indicated there was no significant difference among populations at the 5% level (based on Tukey’s multiple comparison test)

The *Wolbachia* density in the double-infection lines varied with the population (Fig. 3). In the line established from the ZJ population, the *Wolbachia* density was significantly higher than that in the lines from the MS, NJ, and NT populations (*F*_4,14_ = 8.832, *P* = 0.003).

**Fig. 4:**
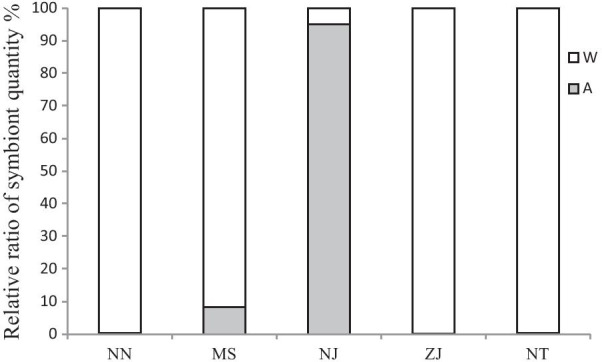
Relative ratio of *Wolbachia* and *Arsenophonus* in the double-infection lines of *N. lugens* varied among geographic populations

The relative ratio of *Wolbachia* and *Arsenophonus* quantity in the double-infected lines of *N. lugens* also varied with the geographic population (Fig. 4). In the double-infected lines from the NN, ZJ and NT populations, the ratio of *Wolbachia* quantity was nearly 100% while that of *Arsenophonus* quantity was all less than 0.4%; however, in the line from the NJ population, *Arsenophonus* was the dominant symbiont and its ratio was 91.7%, and in the double-infected line from the MS population, the ratio of *Arsenophonus* quantity was 8.3%.

## Discussion

When different symbionts are simultaneously present within the same host, interactions between them will take place, which might affect the dynamics of the microbial population. The interaction of the common endosymbiont *Wolbachia* with other symbionts has received increasing attention. An asymmetrical interaction has been found between *Wolbachia* and *Spiroplasma* [7]. Our aim in this study was to test whether interactions between *Wolbachia* and another popular symbiont, *Arsenophonus*, in the same host could affect the titre of the symbionts. We established 5 double-infected lines from different natural populations of *N. lugens*, and they were stable co-infections.

Previous studies have shown that the brown planthopper population from Laos was extensively infected by *Wolbachia* or *Arsenophonus* and the two bacteria may be exclusive in each host individual [26]. We found that *Arsenophonus* and *Wolbachia* could coexist in the same individual of brown planthopper in all the tested populations from China and differences among populations might result from differences in population resources.

The double-infection incidence of *Wolbachia* and *Arsenophonus* in brown planthopper varied with the geographical populations in China. In the ZJ population, the double-infection incidence was the highest, with half of the individuals simultaneously harbouring *Wolbachia* and *Arsenophonus*, whereas in the NJ and MS populations, less than 3% were infected with the two symbionts. The variance in double infection has been found in small brown planthopper, with a significantly higher co-infection incidence of *Wolbachia* and *Serratia* observed in the buprofezin-resistant strain compared with that of the buprofezin-susceptible strain [18].

Interactions between coexisting symbionts may affect infection densities because the symbionts may compete for available resources and space in the host body or they may share the resources and habitats by regulating their own exploitation to avoid damaging the whole symbiotic system [3, 12, 16, 29]. In pea aphids, the density of the primary symbiont *Buchnera aphidicola* is depressed when the insect is co-infected with *Serratia symbiotica* [16] or *Rickettsia* [29]. An antagonistic interaction between *Hamiltonella* and *Cardinium* has also been found in *Bemisia tabaci*, and the density of *Cardinium* increased across time and led to a decrease of *Hamiltonella* density [40]. Asymmetrical interactions have been found between the reproductive parasites *Spiroplasma* and *Wolbachia* in *Drosophila melanogaster* in which the population of *Wolbachia* organisms was affected by *Spiroplasma* while the population of *Spiroplasma* was not affected by *Wolbachia* [7]. Other than the interaction between different species of symbionts, interactions are also observed between different strains of the same symbiont. When multiple *Wolbachia* strains were observed in the same host, the density of each strain was specifically regulated [13, 17], which limited the segregation of symbionts through inefficient transmission by maintaining a sufficiently high density of each symbiont [4].

In our study, we found that in brown planthopper, co-infection with *Wolbachia* did not negatively affect eh *Arsenophonus* population and did not lead to lower net bacterial densities. In addition, the relative ratio of *Wolbachia* and *Arsenophonus* quantity in the double-infected lines of *N. lugens* varied with the geographic population. In the double-infected lines from the NN, ZJ, NT and MS populations, *Wolbachia* was the dominant symbiont, whereas in the double-infected line from the NJ population, *Arsenophonus* was the dominant symbiont and had a significantly higher density than that of *Wolbachia*. The difference in *Arsenophonus* density among lines might be related to the period of maintenance in the lab because the NJ population has been maintained for more than 14 years before investigation, which is at least 7 years longer than the other populations. This longer period of maintenance may possibly benefit the accumulation of *Arsenophonus.*

*Wolbachia* can provide protection against environmental stress, including RNA viruses and insecticides [11, 18, 19, 36], and this genus also confers certain fitness benefits to their hosts [22, 37]; however, *Wolbachia* can also have deleterious effects on the life history of their hosts [5, 9]. *Arsenophonus* was also found to provide protection against environmental stress, such as protection against the entomopathogenic fungi *Metarhizium anisopliae* [44], although it also induced negative effects on their hosts, such as decreasing the chemical insecticide (imidachloprid) resistance of rice brown planthopper [25]. Co-infection of *Wolbachia* and *Arsenophonus* is stable in brown planthopper, which raises the question of how these genera evolve and the effect that they have on the phenotype of their host.

## Conclusions

Interactions of *Wolbachia*, the most common symbiont, with *Arsenophonus,* another widely distributed symbiont in nature has not been reported previously. Present study indicated that *Wolbachia* and *Arsenophonus* could coexist in *N. lugens*, and the co-infection incidence varied with the geographic populations. Antagonistic interactions were not observed between *Arsenophonus* and *Wolbachia*, and *Wolbachia* was the dominant symbiont in most populations.

## Data Availability

The datasets used and analysed during the current study are available from the corresponding author on reasonable request.
